# Short-term changes in human metabolism following a 5-h delay of the light-dark and behavioral cycle

**DOI:** 10.1016/j.isci.2024.111161

**Published:** 2024-10-11

**Authors:** Alan Flanagan, Leonie C. Ruddick-Collins, Barbara Fielding, Benita Middleton, Johanna von Gerichten, Michael Short, Victoria Revell, Jeewaka Mendis, Claus-Dieter Mayer, Peter J. Morgan, Alexandra M. Johnstone, Jonathan D. Johnston

**Affiliations:** 1 Section of Chronobiology, Faculty of Health and Medical Sciences, University of Surrey, Guildford, Surrey GU2 7XH, UK; 2 Section of Molecular Medicine, Food and Macronutrients, Faculty of Health and Medical Sciences, University of Surrey, Guildford, Surrey GU2 7XH, UK; 3 The Rowett Institute, University of Aberdeen, Foresterhill, Aberdeen, Scotland AB25 2ZD, UK; 4 Department of Chemical and Process Engineering, Faculty of Engineering and Physical Sciences, University of Surrey, Guildford, Surrey GU2 7XH, UK; 5 Surrey Sleep Research Centre, Faculty of Health and Medical Sciences, Guildford, Surrey GU2 7XP, UK; 6 Surrey Clinical Trials Unit, Faculty of Health and Medical Sciences, Guildford, Surrey GU2 7XP, UK; 7 Biomathematics and Statistics Scotland, University of Aberdeen, Foresterhill, Aberdeen, Scotland AB25 2ZD, UK

**Keywords:** biological sciences, physiology, human metabolism

## Abstract

Experimental inversion of circadian and behavioral rhythms by 12 h adversely affects markers of metabolic health. We investigated the effects of a more modest 5-h delay in behavioral cycles. Fourteen participants completed an 8-day in-patient laboratory protocol, with controlled sleep-wake opportunities, light-dark cycles, and diet. The 5-h delay in behavioral cycles was induced by delaying sleep opportunity. We measured melatonin to confirm central circadian phase, fasting markers and postprandial metabolism, energy expenditure, subjective sleepiness, and appetite, throughout the waking period. After the phase delay, there was slower gastric emptying at breakfast, lower fasting plasma glucose, higher postprandial plasma glucose and triglycerides, and lower thermic effect of feeding. Any changes were abolished or attenuated within 48–72 h. These data extend our previous findings, which showed no time-of-day effect in healthy adults on daytime energy expenditure or thermic effect of feeding when accounting for circadian variation in resting metabolic rate.

## Highlights

• 5-h circadian desynchrony disrupted metabolic physiology and subjective appetite
• Resting energy expenditure did not change across the waking period
• Gastric emptying and postprandial responses were all disrupted
• Metabolic disturbance was largely reversed within 48–72 h of the desynchrony

## Graphical abstract

**Figure:**
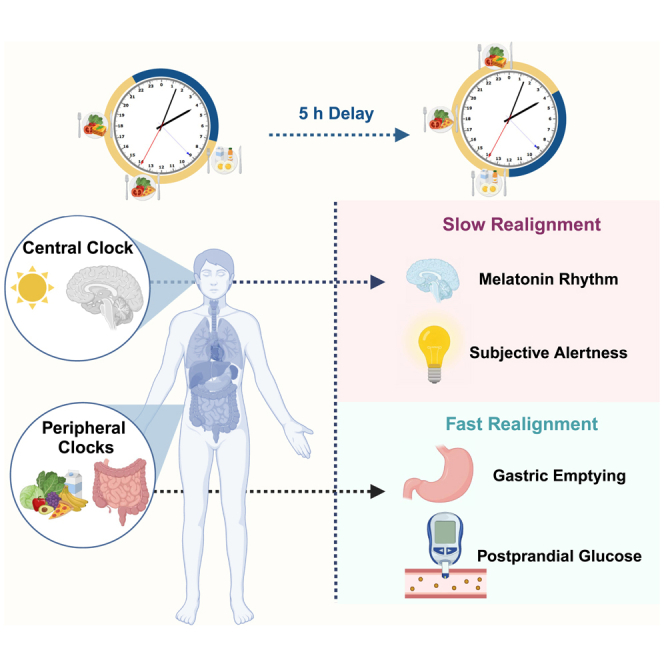

## Introduction

Circadian rhythms have a period of approximately 24 h and are important for multiple aspects of physiology, including cardio-metabolic health.^1^^,^^2^ They are generated by endogenous clocks, and synchronized to external environmental time cues, called zeitgebers.^3^ In mammals, the “central” clock is located within the suprachiasmatic nuclei (SCN) of the hypothalamus and is synchronized by the environmental light-dark cycle.^4^^,^^5^ Physiological outputs from the SCN synchronize “peripheral” clocks throughout the rest of the body. Peripheral clocks regulate metabolism and other key tissue functions and can also entrain independently of the SCN to other environmental inputs, in particular meal timing.^6^^,^^7^^,^^8^

The evolving field of chrono-nutrition combines traditional circadian research with mealtime and diet composition, to explore the impact of feeding and fasting on body weight control and biomarkers of health.^2^^,^^9^^,^^10^ Delaying meals by 5 h within a fixed light-dark schedule induced a delay of 5–6 h in rhythmic glucose metabolism^6^ and increased appetite while reducing energy expenditure.^11^ Postprandial metabolism, in particular glucose and insulin responses, follows a 24-h rhythm^12^^,^^13^^,^^14^ with a well-defined endogenous circadian component.^15^^,^^16^^,^^17^ The rate of gastric emptying may be lower in the evening compared to the morning, although evidence is limited.^18^ Human resting metabolic rate (RMR) has been shown to exhibit a circadian rhythm.^19^ Postprandial energy expenditure, in the thermic effect of food (TEF), has also been proposed to vary according to time of day,^20^^,^^21^^,^^22^^,^^23^ although our work indicates that rhythms in postprandial energy expenditure do not exist *per se*, and previous findings are a consequence of circadian RMR.^24^

Another aspect of chrono-nutrition that has received interest is morning-loaded calorie intake (large breakfast and small dinner) compared to evening-loaded intake (small breakfast and large dinner). In a highly controlled 4-week cross-over weight loss trial, morning-loaded calorie intake reduced appetite compared to evening-loaded calorie intake, suggesting that calories ingested in the morning can help control appetite during energy deficit.^25^ Despite this effect on appetite, the distribution of calorie intake across the day did not impact energy metabolism.^25^ However, this study only applied morning measurement of resting energy metabolism and TEF, and monitoring diurnal changes is preferable. Nonetheless, previous studies conducted using a parallel design over 12 or more weeks suggested morning-loaded calorie intake may increase weight loss.^26^^,^^27^

Together, these studies indicate the importance of time to the short- and long-term response to calorie intake. There remains, however, a clear requirement for additional controlled studies in humans to explore mechanisms of daily metabolism. This is especially true for protocols that model real-life scenarios such as circadian desynchrony and the misalignment between internal biological rhythms and the external environment. Most studies of circadian desynchrony to date have employed a 12-h change in environmental/behavioral cycles, but the effect of more moderate desynchrony, as commonly experienced in, e.g., *trans*-meridian flights, is poorly understood. Within a controlled 8-day laboratory protocol, we therefore exposed participants to an acute 5-h delay in light-dark, sleep-wake, and feed-fast cycles. We hypothesized that circadian desynchrony the day after the 5-h delay would impair daytime energy metabolism, specifically that thermic response to food would be lower after the 5-h phase delay, and only partially return to baseline over the following 5 days.

## Results and discussion

**Figure 1:**
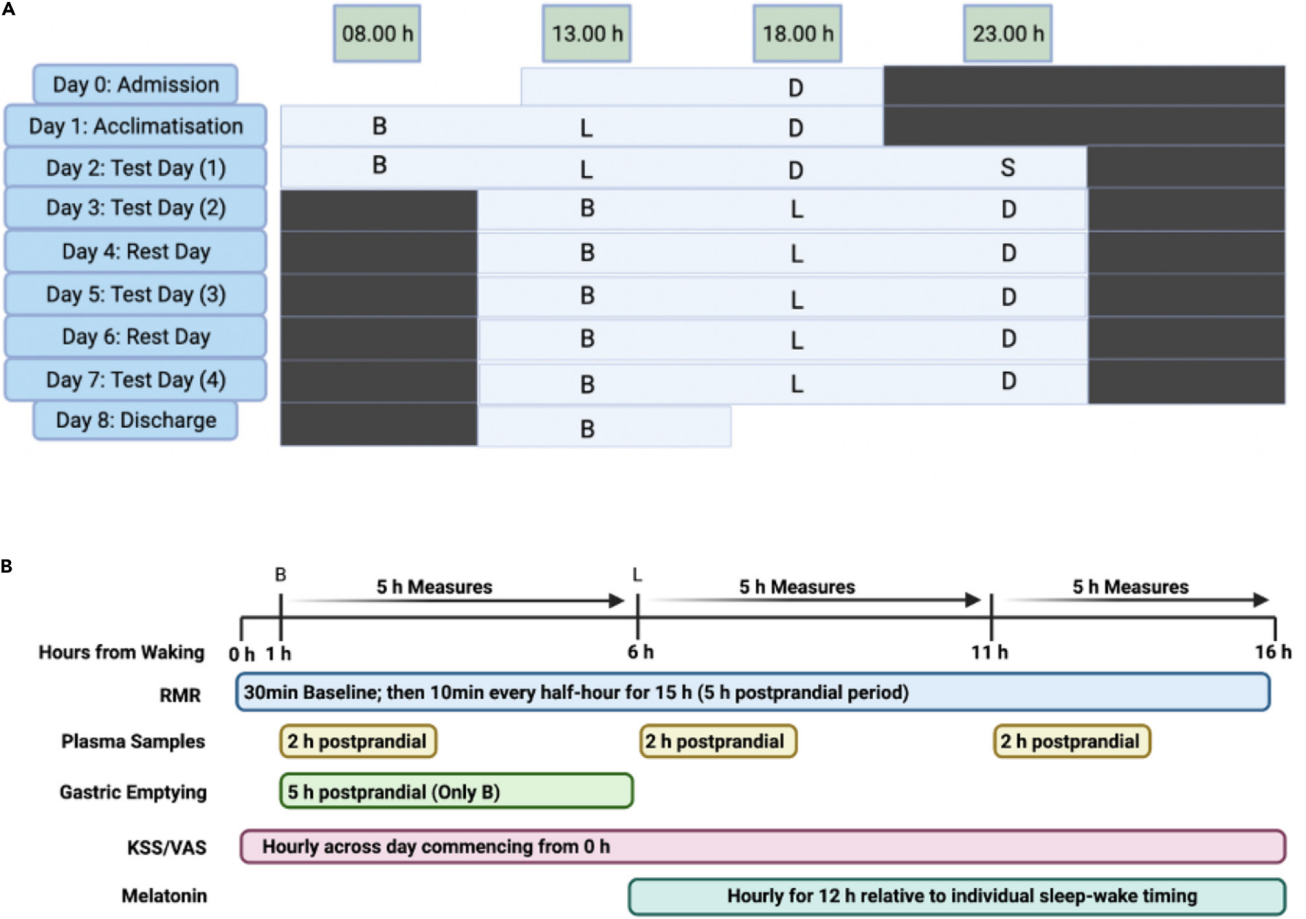
Study protocol and experimental measures (A) Illustration of the phase shift protocol with example of a participant on a 23:00- to 07:00-h sleep schedule (waking at 07.00 h and sleep time at 23.00 h). Light bars indicate lights on and wakefulness. Black bars indicate lights off and sleep opportunity. B, breakfast; L, lunch; D, dinner; S, snack. Meals started 1 h after waking and were separated by 5 h. On Day 2, lights-on was extended by 5 h to induce the phase shift. On Day 2, a snack was provided at 23.00 h to maintain a consistent 16-h fasting period to breakfast on Day 3. Lunch and dinner on Day 2 are aligned in clock time to breakfast and lunch after the phase shift to permit comparisons between meals occurring at the same clock time during aligned versus misaligned conditions. (B) Test days (Day 2, Day 3, Day 5, and Day 7) protocol. Lights-on in the individual sleep laboratories occurred 1 h before breakfast. Bodyweight was measured following voiding upon waking. Baseline subjective questionnaires (KSS/VAS) and the baseline 30-min resting metabolic rate (RMR) measure were completed; 5-min prior to breakfast, baseline breath samples were taken for gastric emptying in addition to baseline fasted blood samples. Breath samples for gastric emptying were taken after breakfast at 15, 30, 45, 60, 75, 90, 120, 150, 180, 240, and 300 min postprandial. Blood samples were taken 15, 30, 60, 90, and 120 min in each postprandial period for glucose, insulin, and triglycerides. Melatonin sampling began in the afternoon ∼11.5 h before habitual bedtime, and 12 samples were taken over 11.5 h. Questionnaires were completed every hour across the waking period. Meals were initiated 1 h after waking, with lunch and dinner separated by 5 h, respectively.

Participants arrived at the laboratory on Day 0 and were admitted following confirmation of adherence to the pre-lab routine by actigraphy. Using a phase-shift protocol, the behavioral cycles of participants including sleep-wake timing, light exposure, and meal timing were delayed by 5 h, beginning on the evening of Day 2 into Day 3 (Figure 1A).^10^ To maintain a consistent fasting period between last energy intake and the test breakfast, a supper was provided 5 h after dinner on the evening of Day 2 (∼23 h). Participants remained on the delayed behavioral/environmental cycle for 5 days to monitor adaptation of biological rhythms to the new schedule. The phase delay had the effect of acutely misaligning the timing of delayed behavioral and environmental cycles from the endogenous circadian rhythms that would initially remain aligned to the sleep-wake timing and meal timing of the participants before the phase delay. It also preserved a nightly 8-h sleep opportunity, to minimize any acute effect of sleep curtailment. The protocol compared energy expenditure, postprandial metabolic responses, and melatonin onset timing throughout the experiment, as follows: (a) Circadian rhythms aligned to behavior cycles (Day 2); (b) acute misalignment of circadian rhythms following 5-h delay in behavioral cycles (Day 3); (c) adaptation of circadian rhythms to the delayed behavioral cycles (Day 5 and Day 7) (Figures 1A and 1B).

Fourteen participants (8 male, 6 female) completed the study. Participant characteristics according to sex can be found in Table 1. Mean age of participants was 45.6years (±9.6), and mean BMI was 31.7 kg/m^2^ (±3.0). The study was a fixed-feeding protocol designed to maintain participants at a stable weight. Energy intake tailored to individual requirements, calculated as basal metabolic rate using the Mifflin-St. Jeor equation,^28^ multiplied by a physical activity factor of 1.1–1.3. Breakfast, lunch, and dinner were isocaloric (33.3% of total daily energy intake), and the macronutrient composition of the diets was 20% protein, 35% fat, and 45% carbohydrate (Table S1). Supper on Day 2 contained the same macronutrient composition. Each meal contained a minimum energy content of 2,301 kJ, with individual participant energy requirements being met through the addition of prepared milkshakes in quantities specific to requirements of the individual participant. Meals were initiated 1 h after waking, with lunch and dinner separated by 5 h, respectively. Compliance was assessed by weighing back any leftover foods and changes in bodyweight during the study. Mean weight change during the in-patient study period was −0.6kg (±0.5), and BMI at the end of the study was 31.5 kg/m^2^ (±3.0). Compliance with the diet, assessed by weighing back any leftover foods, was excellent, supported by the <1% change in bodyweight during the in-patient laboratory period.

**Table 1:** Participant characteristics

	Male (*n* = 8)	Female (*n* = 6)
Age [yrs]	48.1 [±7.5]	42.3 [±11.8]
Height [cm]	178.9 [±4.9]	165.7 [±8.3]
Baseline weight [kg]	100.6 [±6.8]	88.2 [±15.7]
Baseline BMI [kg/m^2^]	31.5 [±3.3]	31.9 [±2.9]
Mean energy intake [kcal]	2373 [±220]	2000 [±270]
Weight change [kg]	−0.8 [±0.4]	−0.4 [±0.4]
BMI change [kg/m^2^]	−0.2 [±3.3]	−0.1 [±2.9]

Note: data are presented as mean and standard deviation (SD).

### Rhythms of melatonin, sleepiness, and alertness exhibit gradual realignment to the new behavioral cycle

**Figure 2:**
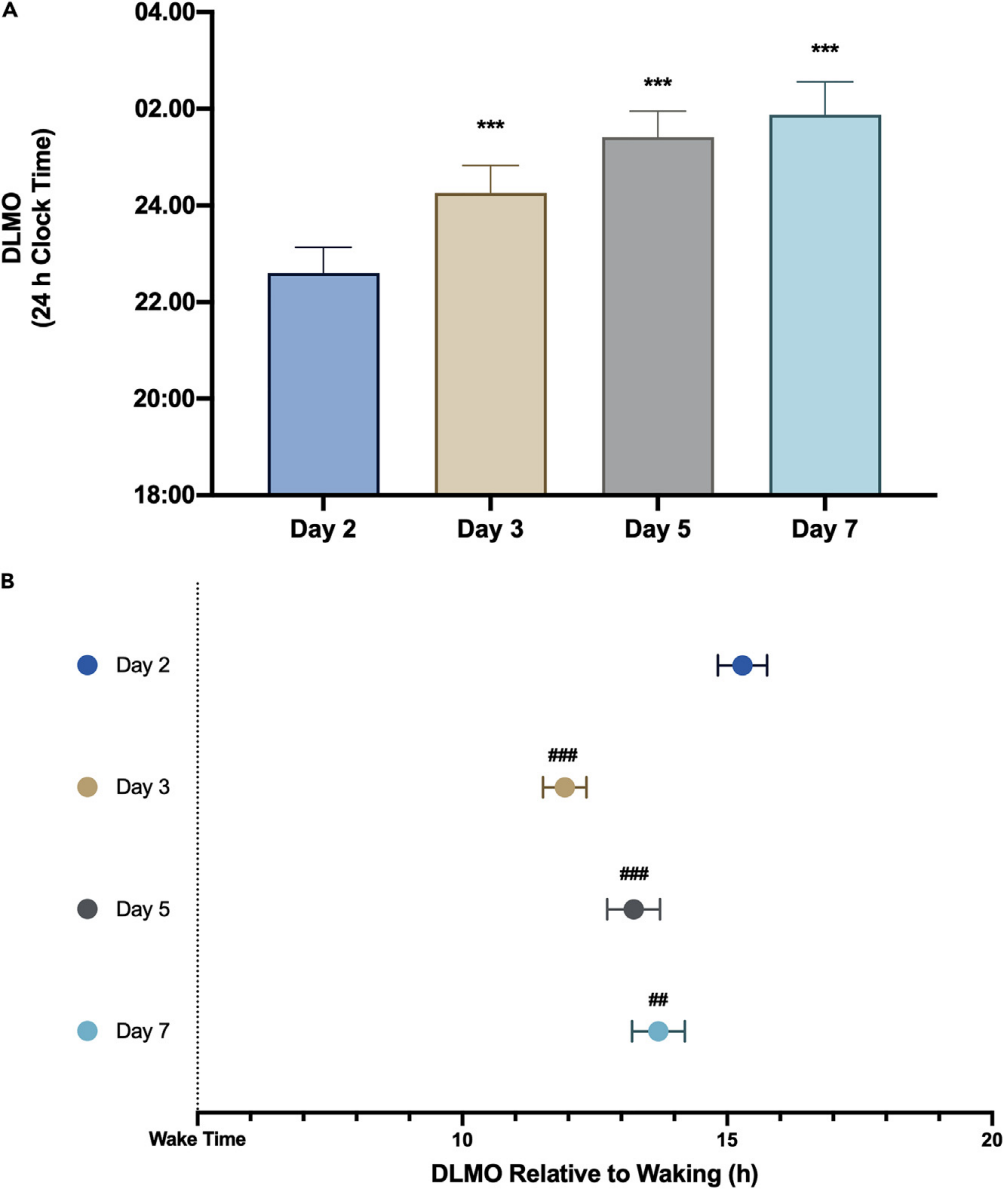
Effects of a 5-h phase delay on melatonin timing (A) Dim-light melatonin onset (DLMO) between study days and (B) time interval between waking and DLMO between study days. ∗∗∗*p* < 0.001 for the difference in DLMO comparing Day 2 to Day 3, Day 5, and Day 7. ###*p* < 0.001 for the difference in DLMO relative to wake time comparing Day 2 to Day 3 and Day 5; ##*p* = 0.005 for the difference in DLMO relative to wake time comparing Day 2 and Day 7. Data are presented as mean ± SEM.

The average pre-shift wake-time and bedtime of participants were 07:21 h (range: 06:30–08:40 h) and 23:10 h (range: 22:30–00:40 h), respectively. Dim-light melatonin onset (DLMO) is the gold-standard marker of the central SCN clock.^29^^,^^30^ We observed a significant effect of study day on measured DLMO (*p* < 0.001). Compared to the pre-phase delay on Day 2, DLMO on Day 3 was 1 h 26 min (*p* < 0.001) later, 3 h 6 min (*p* < 0.001) later on Day 5, and 3 h 38 min (*p* < 0.001) later on Day 7 (Figure 2A; Table S2). Previous research investigating the effects of 6-h westbound flight showed that DLMO delayed by 1.3 h per day on average.^31^ Thus, our DLMO data demonstrated a similar rate of resynchrony as described in previous research.^6^^,^^31^ This change in DLMO occurred despite the participants wearing blue-light-blocking glasses in the second half of the wake period and so receiving less blue light during the phase delay portion of the photic phase-response curve. The change in DLMO, and thus SCN phase, is therefore likely driven by a lack of morning (phase advancing) light, incomplete blockade of light by the glasses, and non-photic signals. The relative contribution of these influences is unclear, but this does not alter the primary findings of the study.

We next considered the time interval between waking and DLMO, in which there was also a significant effect of study day (*p* < 0.001). DLMO occurred at an average of 15 h 18 min after wake time on Day 2. However, compared to Day 2, the DLMO on Day 3 occurred 3 h 33 min (*p* < 0.001) closer to wake time, on Day 5 occurred 1 h 53 min (*p* < 0.001) closer to wake time, and on Day 7 occurred 1 h 12 min (*p* = 0.005) closer to wake time (Figure 2B; Table S2).

**Figure 3:**
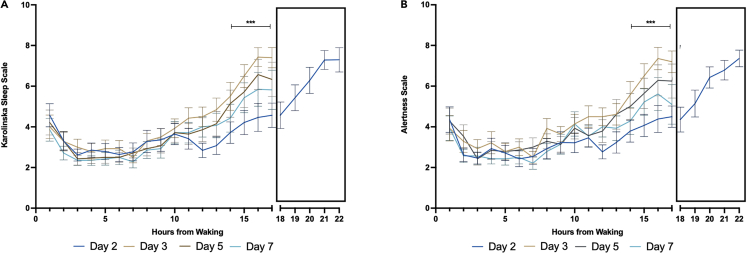
Effects of a 5-h phase delay on daily profiles of sleepiness and alertness (A) Karolinska Sleepiness Scale (KSS) scores. The KSS is a 9-point scale of discreet numbers between 1 and 9 corresponding with a verbal anchor from: 1 “Extremely Alert” to 9 “Fighting Sleep”. (B) Alertness Scale, which contains two verbal anchors and a corresponding discrete number: 1 “Very Alert” to 9 “Very Sleepy” (i.e., higher scores indicate lower alertness). Data are presented as mean ± standard error. ∗∗∗*p* < 0.001 for the difference in KSS and Alertness from 14 to 17 h after waking, compared to the morning, on Days 3, 5, and 7. The truncated X axis represents the additional time points included for the 5-h extended waking period on Day 2 to induce the phase shift. Black = Day 2; Red = Day 3; Green = Day 5; Purple = Day 7. Data are presented as mean ± SEM.

The effect of the 5-h phase delay was also confirmed by the significant changes in subjective sleepiness and decreased subjective alertness that were observed immediately following the phase delay (Figures 3A and 3B). Higher scores on the Karolinska Sleepiness Scale (KSS) indicated higher sleepiness, whereas higher scores on the Alertness scale indicated lower alertness levels. For sleepiness and alertness, there was a significant day-by-time-point interaction, (*p* < 0.001), and a significant main effect of time point (*p* < 0.0001), but no significant main effect of study day (*p* = 0.412). Between 14 and 17 h after waking on Day 3, participants exhibited significantly greater sleepiness (*p* < 0.001) and significantly lower alertness levels (*p* < 0.001) at levels similar to those experienced after 20–22 h of wakefulness on Day 2. Between Day 3 to Day 7, temporal profiles of sleepiness and alertness gradually reverted toward levels similar to those observed on Day 2 (Figures 3A and 3B). The actigraphy data indicated no significant difference in sleep efficiency, actual wake time (as a percentage of total sleep time), or actual sleep time (as a percentage of total sleep time) (Figure S1).

### Energy expenditure is unaffected, whereas postprandial thermic effect of feeding adapts with the central clock, following a 5-h phase delay

**Figure 4:**
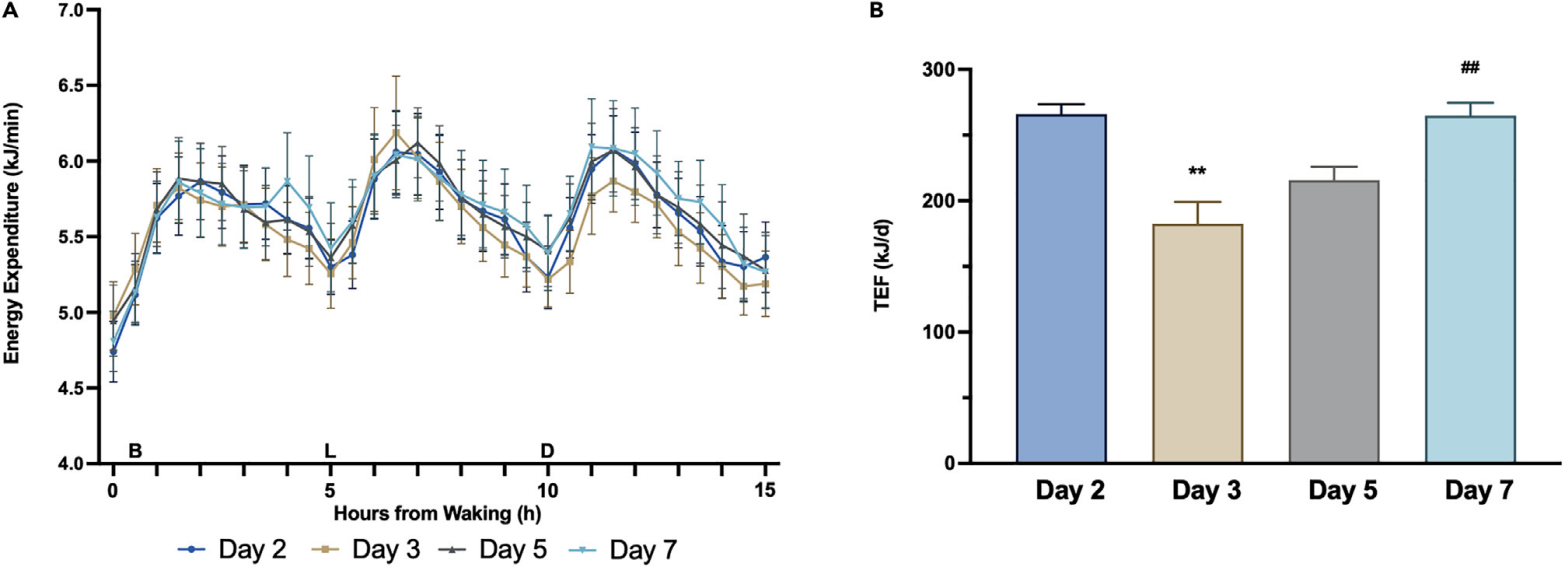
Whole-day energy expenditure and thermic effect of food responses (A) Time course of whole-day (16.5 h) energy expenditure (kJ/min) between study days (Black = Day 2; Red = Day 3; Green = Day 5; Purple = Day 7). (B, L, and D) indicate the timing of breakfast, lunch, and dinner, respectively, relative to energy expenditure measurements. The fasting resting metabolic rate measure was conducted for 30 min upon waking, which is represented as 0 on the X axis. Data are presented as mean and standard error. (B) Thermic effect of feeding [kJ/d] between test days. Data are presented as bars with mean and standard error. **∗∗***p* < 0.01 for the difference in TEF comparing Day 3 to Day 2; ##*p* < 0.01 for the difference in TEF comparing Day 7 to Day 3. Data are presented as mean ± SEM.

We have recently reported findings from a controlled hypocaloric dietary intervention demonstrating that morning versus evening energy load does not influence energy metabolism in humans, when measuring daily RMR or TEF in response to varying meal energy content in the morning period.^25^ In our previous study, we measured basal energy expenditure (RMR) and post-prandial energy metabolism using indirect calorimetry only at breakfast. In the present study, we also utilized indirect calorimetry to measure energy metabolism in a laboratory setting throughout the whole waking period, to examine time-of-day effects. Our present study also has the advantage of extended 5-h postprandial TEF measures, which, factoring in the energy content of meals, likely captures the majority of the TEF response.^32^ There was no difference in whole-day waking energy expenditure (EE) (*p* = 0.368) or fasting RMR (*p* = 0.145) between days (Figure 4A; Table S2). Thus, the present study corroborates and extends our previous findings,^25^ by demonstrating no differences in waking energy expenditure measured robustly measured over 15.5 h of the 16-h waking period. This finding is also consistent with Ravussin et al.,^33^ who found no differences in 24-h energy expenditure measured in a respiratory chamber comparing front-loaded energy intake within a time-restricted 6-h period to isoenergetic meals consumed over 12 h.

Previous studies using indirect calorimetry to measure postprandial TEF responses have suggested ∼2-fold higher TEF following breakfast compared to dinner.^20^^,^^21^^,^^22^ Our recent model proposed in Ruddick-Collins et al.^24^ suggests that underlying circadian variance in RMR likely explains any apparent differences in postprandial TEF responses between meals at different times of day. The present study showed no day-by-meal interaction (*p* = 0.964) or main effect of meal for TEF responses (*p* = 0.208). However, there was a main effect of study day (*p* < 0.001), i.e., the total daily (sum of all meals) overall TEF response. Total daily TEF immediately following the phase delay (Day 3) was 83.3 kJ (*p* = 0.001) lower compared to Day 2, before a gradual increase such that by Day 7, TEF responses were near-identical to the baseline pre-phase shift Day 2, i.e., total TEF on Day 7 was 82.2 kJ (*p* = 0.001) higher compared to Day 3 (Figure 4B; Table S2). These data are in agreement with our proposed model of circadian RMR.^24^ The change in total daily TEF observed in this study, without any significant difference in TEF responses to individual meals, likely reflects central circadian control of RMR rather than altered TEF *per se*, given circadian rhythmicity in RMR is tied to the peak and nadir of core body temperature (CBT),^19^ and would be expected to shift gradually in line with the SCN clock.^34^ Consequently, TEF is likely to be of limited importance for influencing energy expenditure in favor of enhanced metabolic capacity at any time of day and in the context of weight management.

### Gastric emptying after breakfast is delayed in response to a 5-h phase delay but exhibits rapid adaptation to the new behavioral cycle

**Figure 5:**
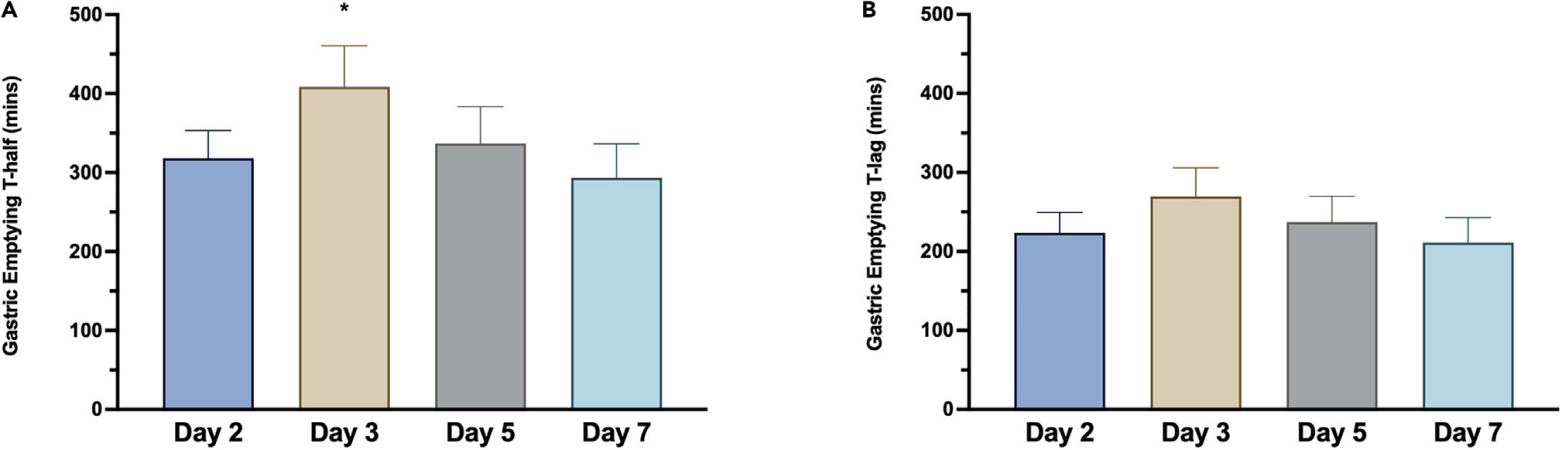
Effects of a 5-h phase delay on gastric emptying (A) Gastric emptying *T-½* between study days. (B) Gastric emptying *T-Lag* between study days. Data are presented as bars with mean and standard error. **∗***p* < 0.05 for the difference in *T-½* comparing Day 3 to Day 2. Data are presented as mean ± SEM.

With the use of stable isotope tracers, gastric emptying (GE) rate may be quantified as the half-time, i.e., the time taken for 50% of the meal to empty the stomach, and the time-lag, i.e., the time point at which the rate of excretion of the tracer reaches its maximum.^35^^,^^36^^,^^37^ Here, we assessed gastric emptying after breakfast each day by the ^13^C octanoic acid breath test.^38^^,^^39^ We show a significant effect of the 5-h phase delay on the GE half-time (T-½) (*p* = 0.038), with post-hoc tests revealing that the T-½ immediately following the phase delay (Day 3) was delayed by 90.3 min (*p* = 0.037) compared to the pre-shift (Day 2) (Figure 5A). There was no significant effect of the phase delay on the gastric emptying time-lag (*p* = 0.155) (Figure 5B). Our data reveal that 5-h misalignment causes an acute effect on gastric emptying in response to the breakfast meal, before adaptation to the new behavioral cycle. A circadian rhythm in gastric emptying has previously been shown, with more rapid emptying in response to identical meals at 8 a.m. versus 8 p.m..^18^ It is possible that the lower gastric emptying half-time following breakfast at 1 p.m. on Day 3 compared to breakfast at 8 a.m. on Day 2 reflects the acute misalignment of the first daily meal relative to this underlying circadian variance in gastric emptying rate. However, as we did not measure gastric emptying in response to lunch, it is not possible from our data to determine whether the difference also may relate to other factors, such as extended wakefulness between Day 2 and Day 3.

### Fasting and postprandial glucose, insulin, and triglyceride concentrations exhibit limited responses to a 5-h phase delay

**Figure 6:**
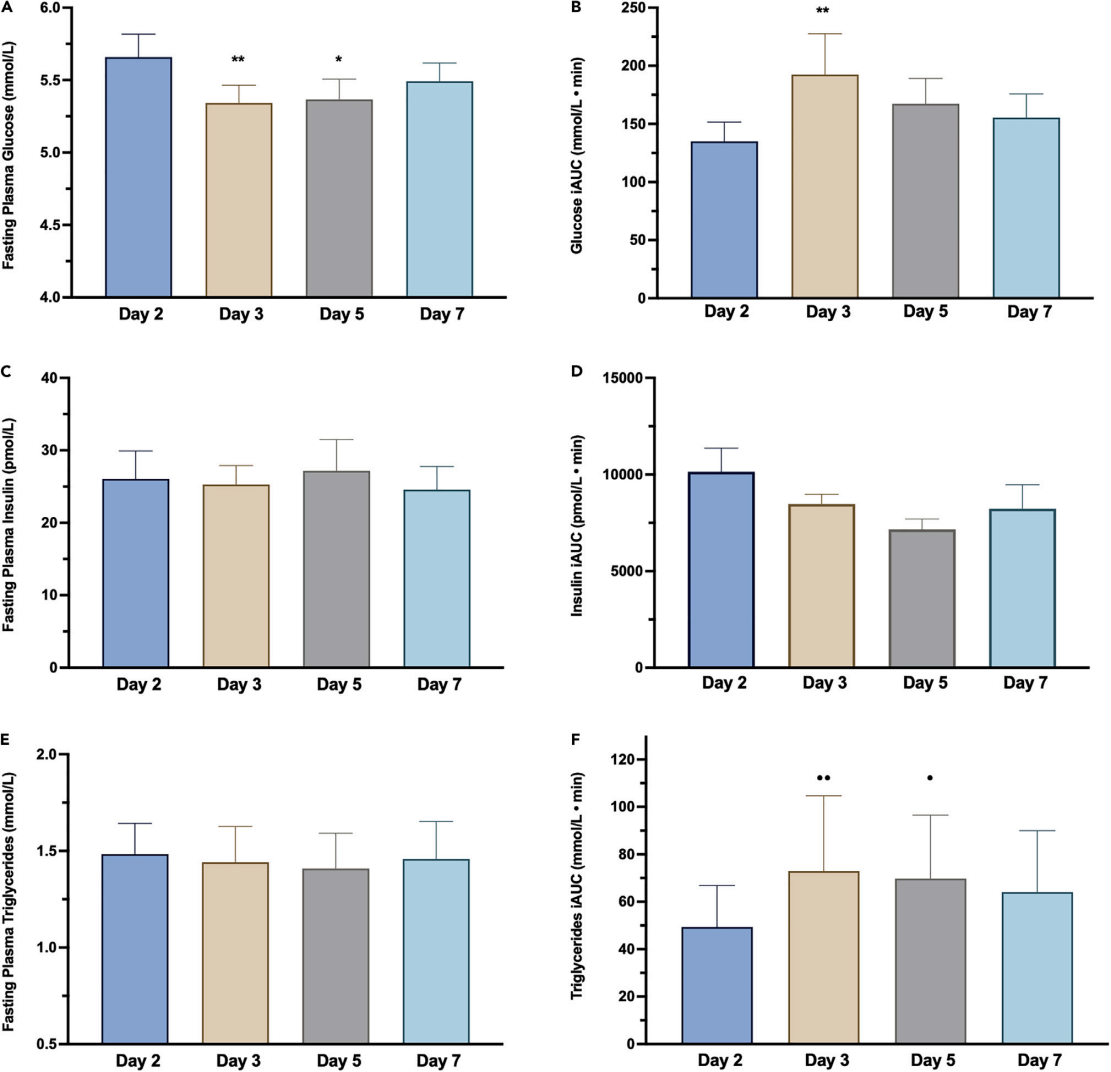
Effects of a 5-h phase delay on fasting and postprandial metabolism (A) Fasting plasma glucose between study days. ∗∗*p* < 0.01 for the difference in fasting glucose comparing Day 3 to Day 2; ∗*p* < 0.05 for the difference fasting glucose comparing Day 5 to Day 2. (B) Postprandial plasma glucose incremental area under the curve (iAUC; mmol/L · min) for the main effect of day. **##***p* < 0.01 for the difference in plasma glucose iAUC on Day 3 compared to Day 2. (C) Fasting plasma insulin between study days. (D) Postprandial plasma insulin iAUC (pmol/L · min) for the main effect of day. (E) Fasting plasma triglycerides (TGs) between study days. (F) Postprandial plasma TG iAUC (mmol/L · min) for the main effect of day. **⋅⋅***p* < 0.01 for the difference in iAUC on Day 3 compared to Day 2 and ⋅ *p* < 0.05 for the difference in on Day 5 compared to Day 2. Data are presented as mean ± SEM.

Higher morning fasting glucose levels compared to the afternoon have been shown in healthy, non-diabetic individuals similar to the participants in our study.^40^^,^^41^ Our data showed a significant effect of day on fasting plasma glucose levels (*p* = 0.003), with post-hoc comparisons showing a 0.31 mmol/L (*p* = 0.005) lower fasting glucose on Day 3 and 0.29 mmol/L (*p* = 0.011) lower fasting glucose on Day 5 when measured at ∼13.00 h following the phase delay, compared to when measured at ∼08.00 h before the phase delay (Figure 6A; Table S2).

There were no significant day-by-meal interactions for postprandial glucose iAUC (*p* = 0.905), but a significant main effect of study day (*p* = 0.005) and a significant main effect of meal (*p* = 0.006). For the main effect of day, total daily postprandial glucose iAUC between days was 57.4 mmol/L⋅min higher on Day 3 compared to Day 2 (*p* = 0.003) (Figure 6B; Table S2). For the main effect of meals, postprandial glucose iAUC was higher on average following breakfast compared to dinner (*p* = 0.004) (Table S2; Figure S2). This is likely due to differences in meal composition as, although our meals were matched for energy and macronutrient content and meals between study days (i.e., breakfast on each study day) was the same, the specific foods at each meal (i.e., breakfast, lunch, and dinner) differed and the glycemic index (GI) and/or glycemic load (GL) were not considered. Nevertheless, the fact that the between-day postprandial glucose iAUC differences showed glucose remaining elevated above the pre-phase delay baseline levels is consistent with the overall effects of later meal timing, relative to SCN phase, on plasma glucose levels.^12^^,^^14^^,^^42^ There was no significant day-by-meal interaction effect for plasma insulin iAUC (*p* = 0.796), but a significant main effect of meal (*p* = 0.008) was evident. For the main effect of meal, post-hoc comparisons indicated that insulin iAUC was 2591.4 pmol/L⋅min (*p* = 0.007) lower after dinner compared to breakfast (Table S1; Figure S2), reflecting the higher postprandial glucose iAUC.

Glucose was also measured with continuous glucose monitoring (CGM), which recorded interstitial glucose levels in 5-min intervals across the day and was used to calculate the Mean Amplitude of Glycaemic Excursions (MAGE; the average amplitude of glucose fluctuations with a magnitude of greater than 1 SD) and the Continuous Overall Net Glycemic Action (CONGA; glucose variability within 1 h segments of a continuous time-series). Data were available for test Days 2, 3, and 5. There was no significant effect of the 5-h phase delay on the MAGE (*p* = 0.196) or on the CONGA (*p* = 0.409) (Table S2).

Circadian variation in circulating levels of triglycerides (TGs) has also been demonstrated under constant routine conditions, characterized by lower basal levels throughout the daytime before increasing and peaking during the biological night.^42^ In response to meal timing, diurnal variation in postprandial TG responses has also been shown with greater elevations in postprandial TG in response to a meal during the biological night compared to the same isocaloric meal during the biological day.^43^ We observed no effect of the intervention on fasting TGs (*p* = 0.262) (Figure 6E, Table S2). For postprandial TG levels, although there was no significant day-by-meal interaction (*p* = 0.242), there were significant main effects of study day (*p* = 0.003) and meals (*p* < 0.001). For the main effect of day, compared to the pre-shift alignment condition, the total daily postprandial TG iAUC on Day 3 was 24.9 mmol/L⋅min (*p* = 0.003) higher and was 20.4 mmol/L⋅min (*p* = 0.026) higher on Day 5 (Figure 6F; Table S2). For the main effect of meal, compared to breakfast, TG iAUC following lunch was 89.4 mmol/L⋅min (*p* < 0.001) higher and 55.5 mmol/L⋅min (*p* < 0.001) higher following dinner (Table S2; Figure S2). Taken together, our data are consistent with previous research demonstrating that mean postprandial TG concentrations are higher at night than during the day.^43^

### Subjective hunger, fullness, and appetite are unaffected by a 5-h phase delay

We assessed subjective measures of hunger, appetite, and fullness using visual analogue scale (VAS), every hour from waking for 16 h during the test days (Figure S3). We found no significant differences in subjective hunger (*p* = 0.992), fullness (*p* = 0.982), or appetite (*p* = 0.990), between days. Previous laboratory studies have indicated that rhythms in subjective hunger and appetite have an endogenous circadian component, peaking in the biological evening, independent of the behavioral cycle and food intake.^6^^,^^44^ We have recently shown that earlier, morning-loaded temporal distribution of energy improves appetite and lowers appetite hormones during hypocaloric dieting.^25^ In a recent laboratory study that delayed meals by 5 h (similar to our present study), such that breakfast, lunch, and dinner occurred 5 h, 9h, and 13 h, respectively, after wake time, the later temporal meal timing increased waking subjective appetite and dysregulated hunger and energy balance hormones.^11^ Using a similar design, Wehrens et al.^6^ did not detect significant effects of mealtimes delayed by 5 h but did show lower morning hunger levels under constant routine conditions. The overall weight of data suggests some temporal variation in subjective hunger and appetite, and the lack of any observed effect in our present study may be due to design and analytical differences between our study and previous research.

### Limitations of the study

The findings from this metabolically healthy sample may not be generalizable to population groups with elevated cardio-metabolic risk factors, which could potentially exhibit more impaired metabolic responses to a 5-h phase delay. Although our participants were metabolically healthy, they represent a different demographic to most human physiology studies. However, this move away from studying healthy young adults is important for the field of human physiology. We have not analyzed our data separately for male versus female participants as the study was not powered to detect sex effects. Although our meals were matched for energy content, it is also possible that the differences in the GI of the meals, particularly breakfast, may have influenced the postprandial glucose and insulin responses observed in our study. Due to the existing complexity and therefore participant burden within the protocol, we did not include objective measures of sleep (e.g., polysomnography) or measure energy metabolism during the sleep period. As in any circadian desynchrony study, altered sleep timing may alter sleep quality. Nonetheless, we provided participants with an 8-h sleep opportunity each day to minimize such effects, so it is unlikely that there was a substantive influence of sleep alteration on our data.

### Conclusions

In response to our 5-h phase delay protocol, some markers of daily metabolism, in particular gastric emptying and total postprandial glucose levels, exhibited acute change following the 5-h phase delay. However, these markers reverted to pre-misalignment values as DLMO, the gold-standard marker of the SCN clock, synchronized to the new behavioral cycle. We confirm and extend our recent research challenging assumptions regarding the time-of-day effect on energy metabolism.^24^ Together, this body of work suggests limited importance of meal timing on TEF. This study represents an important refinement of previous proof-of-concept studies to model physiological challenges experienced in the real world.

## Resource availability

### Lead contact

Further information and requests for resources and reagents should be directed to and will be fulfilled by the lead contact by Professor Jonathan D Johnston, Section of Chronobiology, School of Biosciences, Faculty of Health and Medical Sciences, University of Surrey, Guildford, Surrey GU2 7XH, UK (j.johnston@surrey.ac.uk).

### Materials availability

This study did not generate new unique reagents.

### Data and code availability

• All data reported in this paper will be shared by the lead contact upon request.
• This paper does not report any original code.
• Any additional information required to reanalyze the data reported in this paper is available from the lead contact upon request.

## Acknowledgments

This work was funded by 10.13039/501100000265MRC grant (MR/P012205/1 to A.M.J., J.D.J., and P.J.M.). We thank staff of the Surrey Clinical Research Facility; L. Bamford for measurement of metabolite concentrations on the Pentra C400 Clinical Chemistry Analyzer; N. Jackson for assistance with the isotopic enrichments at the Stable Isotope Mass Spectrometry Unit, University of Surrey; F. Shojaee-Moradie for assistance with ELISA; A. Collins, H. Biyikoglu, and R Manders for assistance with operating GEMs; and P. Jefcoate and A. Brealy for assistance with meal preparation. Finally, we thank D van der Veen for helpful discussions during manuscript preparation.

## Author contributions

Conceptualization, A.M.J., J.D.J., P.J.M., and L.R.C.; methodology, A.M.J., J.D.J., P.J.M., L.R.C., B.F., J.v.G., M.S., J.M., and C.D.M.; formal analysis, J.M., A.F., J.v.G., M.S., and C.D.M.; investigation, A.F., L.R.C., and V.R.; writing—original draft, A.F. and L.R.C.; writing—review & editing, all authors; funding acquisition, A.M.J., J.D.J., and P.J.M.; supervision, J.D.J., B.F., A.M.J., P.J.M., and L.R.C.

## Declaration of interests

J.D.J. has collaborated with Nestle ´and has undertaken consultancy work for Kellogg’s and International Flavors and Fragrances (IFF). A.M.J holds a voluntary position at British Nutrition Foundation, London, as an Advisory Committee member.

## STAR★Methods

### Key resources table

**Table:** 

REAGENT or RESOURCE	SOURCE	IDENTIFIER
**Biological samples**		

Human Blood and Saliva Samples	This study	N/A

**Chemicals, peptides, and recombinant proteins**		

13^c^ Octonoic Acid	CK Isotopes Limited	DLM-619

**Critical commercial assays**		

Insulin ELISA Assays	DRG International GmbH	Cat# EIA-2935
ABX Pentra Glucose Assays	Horiba Instruments Ltd.	Cat# A11A01668
ABX Pentra Triglyceride Assays	Horiba Instruments Ltd.	Cat# A11A01640
Direct Saliva Melatonin RIA	NovoLytiX GmbH	Cat# RK-DSM2

**Software and algorithms**		

IBM SPSS, Version 27.0	Armonk, NY, USA: IBM Corp	https://www.ibm.com/products/spss-statistics
SAS, Version 9.4	NC, USA: SAS Institute Inc	https://www.sas.com/en_us/software/stat.html
GraphPad Prism, Version 9.1	GraphPad Software 2021, La Jolla California, USA	https://www.graphpad.com/
WinDiets software	Professional Version, Robert Gordon University, Aberdeen, UK, 2017	https://www.rgu.ac.uk/windiets
CareLink iPro Therapy Management Software (CGMS; web-based system)	Medtronic Limited, Hertfordshire UK	https://carelink.minimed.eu/ipro/

**Other**		

RMR and ventilated hood	GEM Nutrition	http://www.gemnutrition.co.uk/
Continuous Glucose Monitors (iPro2 system)	Medtronic Limited, Hertfordshire UK	https://www.medtronic.com/uk-en/index.html

### Experimental model and study participant details

#### Participants

All study procedures were reviewed and given a favourable ethical opinion by the University of Surrey Ethics Committee [UEC/2018/041/FHMS (CRC382)]. Fourteen participants (8 male, 6 female) completed the study in a single experimental group [Table 1]. All participants were of White British ethnicity. All participants provided written informed consent prior to participation in the study. Eligibility criteria included adult males and females aged 20–60 years, body mass index (BMI) of 27–40 kg/m^2^ inclusive, habitual 6–9 h sleep per night, and otherwise healthy. Exclusion criteria included prescription or nutritional supplement use known to affect sleep, extreme chronotype (Horne-Östberg score <30 or >70; habitual bedtime outside 22:00-01:00), any history of sleep or circadian disorders, history of metabolic disease, or current smokers. As a condition of entry to the laboratory study, participants were required to follow a 1-week baseline run-in period, maintaining a consistent and habitual sleep-wake timing and consistent spacing between breakfast, lunch, and dinner, to be consumed 1 h, 6 h, and 11 h after waking, respectively. Participants were also required to obtain 15 min of natural, outdoor light exposure within 90 min of waking.

To confirm adherence, participants were required to call the University of Surrey Clinical Research Facility [CRF] voicemail within 10 min of going to bed and waking up. In addition, participants were also provided with two actiwatches (Cambridge Neurotechnology, Cambridge, UK), which recorded activity levels and environmental light levels; one actiwatch was worn on the non-dominant wrist to record activity, while the other was worn around the neck to monitor light exposure near eye level. The actigraphy data was downloaded on Day 0 when participants presented to the CRF for admission to the study, to ensure compliance with the baseline sleep-wake schedule. Compliance confirmed by actigraphy was a condition of admission to the laboratory phase of the study.

### Method details

#### Laboratory study design

We evaluated the effect of a 5 h phase delay on energy expenditure and related endocrine and metabolic pathways in humans. This objective was achieved by conducting a 7-day residential, phase-shift protocol, in which the behavioral cycles of sleep-wake timing, light-dark, and meal timing, were delayed by 5 h between days 2–3 [Figures 1A and 1B].^10^ Throughout the laboratory study, light levels were maintained at 500 lux in the direction of gaze and blue light-blocking glasses were worn for the second half of the lights-on/wake period. The sleep-wake timing for all participants during the in-patient stay were standardised to within 30mins of their habitual sleep-wake timing from the run-in period.

The extension of the light cycle by 5 h between Day 2 and Day 3, coupled with the related delay in the sleep-wake cycle and timing of meals the following day by 5 h, created misaligned timing of cues in light, sleep-wake, and meals, for the circadian system. This “phase delay” or “phase shift” would have the effect of altering the timing of external behaviors [light-dark, sleep-wake, and meals], while internal circadian rhythms would initially remain aligned with the previous timing of behaviors on Day 2. The phase delay also preserved a nightly 8 h sleep opportunity, to minimise any acute effect of sleep curtailment.

Participants arrived at the laboratory on Day 0 and were admitted following confirmation of adherence to the pre-lab routine by actigraphy [Figure 1A]. Participants continued to wear the actiwatch on their non-dominant wrist to provide estimates of sleep-wake parameters derived from the actigraphy data. Day 1 of the protocol served as a habituation day to the study schedule and laboratory conditions maintained at the pre-laboratory sleep-wake and feed/fast cycles, and no testing was conducted on this day. Day 2 of the protocol followed the exact same timing of the feeding/fasting and sleep/wake cycle as Day 1, but included baseline testing for experimental measures.

A 5-h delay in sleep onset and feed-fast/light-dark was induced on the evening of Day 2 into Day 3. To maintain a consistent fasting period between last energy intake and the test breakfast, a supper was provided 5 h after dinner on the evening of Day 2 [∼23.00 h]. Participants remained on the delayed behavioral/environmental cycle for 5 days to monitor adaptation of biological rhythms to the new schedule. The protocol compared energy expenditure, metabolic responses, and melatonin onset timing throughout the experiment, as follows: a) Daily rhythms aligned to behavior cycles [Day 2]; b) Acute misalignment of daily rhythms following 5 h delay in behavioral cycles [Day 3]; c) Adaptation of daily rhythms to the delayed behavioral cycles [Day 5 and Day 7] [Figure 1A].

The study was a fixed-feeding protocol designed to maintain participants at a stable weight. Energy intake tailored to individual requirements, calculated as basal metabolic rate using the Mifflin-St. Joer equation,^28^ multiplied by a physical activity factor of 1.1–1.3. Breakfast, lunch, and dinner were isocaloric [33.3% of total daily energy intake], and the macronutrient composition of the diets was 20% protein, 35% fat, and 45% carbohydrate [Table S2]. Supper on Day 2 was matched to the energy content of other meals and was on average ∼3,123kJ additional energy, with the same macronutrient composition of other meals. Each meal contained a minimum energy content of 2301kJ, with individual participant energy requirements being met through the addition of prepared milkshakes in quantities specific to requirements of the individual participant. Meals were initiated 1 h after waking, with lunch and dinner separated by 5 h, respectively.

#### Basal energy expenditure

Basal energy expenditure (resting metabolic rate, RMR) and the thermic effect of feeding [TEF] were measured using indirect calorimetry [GEM Nutrition, Bath, UK]. RMR was measured for 30-min, within 5-min of waking, to obtain a fasted baseline RMR measure. Thereafter, participants’ RMR was measured for 10-min every half-hour, for 5 h after breakfast, lunch, and dinner respectively. Thus, measures of energy expenditure covered 15.5 h of the 16 h waking period [Figure 1B]. Participants lay in a semi-supine position for the duration of each measurement. RMR was calculated from VO_2_ and VCO_2_ using the Elia and Livesey^45^ equation and measured on a minute-by-minute basis. For the baseline fasted RMR measure, the initial 5-min of the data was excluded, and the RMR calculated from a 15-min moving average with the lowest coefficient of variation [CV]. Postprandial energy expenditure from each subsequent 10-min measurement was determined by calculating consecutive data from a minimum of 5-min with the lowest CV. The TEF was calculated using incremental area under the curve [iAUC] based on a number of methods, reflecting different methodological approaches used in the literature, as described by Ruddick-Collins et al*.*^24^

#### Blood and saliva collection

Blood samples for glucose, lipids, and insulin, were collected immediately prior to each meal via a cannula inserted into the forearm, and then at 15, 30, 60, 90, and 120 min time points in the postprandial period [Figure 1B]. 4.5mL was collected at each time point, and separated, respectively, into 2mL lithium heparin and 2mL potassium EDTA vacutainers. In addition, to assess circadian phase, blood samples for melatonin were collected every hour for 13 consecutive samples, e.g., between 16.00 h and 04.00 h [Figure 1B]. 4mL blood was collected into lithium heparin vacutainers for this. In the event that there were difficulties obtaining a melatonin blood sample, participants provided a 2mL saliva sample collected at the scheduled collection time point. Upon collection of a blood sample, sample tubes were inverted ten times and cooled until centrifugation [1260 x *g* at 4°C for 10-min, within 30-min of collection]. Plasma samples were then stored at −20°C [melatonin] or −80°C [glucose, lipids, insulin], pending analysis. Saliva samples were sealed and stored immediately at −80°C.

#### Endocrine and metabolic measures

Plasma melatonin was analyzed by ^3^H melatonin radioimmunoassay, which has been validated by gas chromatography-mass spectrometry [GCMS], as previously described.^46^ The inter-assay CV was 23.5% at 9.7 pg/mL, 8.4% at 35.5 pg/mL, 9.8% at 73.5 pg/mL, and 10.1% at 136.5 pg/mL. Salivary melatonin was analyzed by the ^125^I melatonin radioimmunoassay.^46^ The inter-assay CV for salivary melatonin was 24.4% at 2 pg/mL and 5.3% at 16.4 pg/mL. Melatonin results were analyzed by calculating the timing of melatonin signal onset, known as dim light melatonin onset [DLMO], an established marker of the endogenous phase of the central SCN clock, as previously described.^29^ 3 pg/mL of salivary melatonin is considered comparable to 10 pg/mL.^29^ Thus, for the present study, DLMO was determined as the time from which melatonin levels rose above of pre-specified absolute threshold of ≥3 and ≥10 pg/mL for salivary and plasma samples, respectively.^29^

Plasma insulin was analyzed by enzyme-linked immunosorbent assay [ELISA] (DRG International, Germany). A standard curve of 0–100uIU/mL was derived from the human insulin standards provided with the ELISA kits. The inter-assay CV was 4.85% at 20.79uIU/mL and 7.28% at 65.98uIU/mL.

Plasma glucose and triglyceride [TGs] concentrations were analyzed by enzyme assays [Pentra C400 Clinical Chemistry Analyzer] (Horiba Medical, UK).^47^^,^^48^ For plasma glucose, the inter-assay CV was 1.61% at 5.23 mmol/L and 1.40% at 14.22 mmol/L For plasma triglycerides, the inter-assay CV was 3.43% at 1.42 mmol/L and 2.75% at 2.57 mmol/L. For plasma glucose, insulin, and plasma TGs, postprandial area under the curve [AUC] and incremental area under the curve [iAUC] were calculated using the trapezoidal rule for the 120min postprandial period, as well as fasting levels. Homeostatic model of insulin resistance [HOMA-IR] was also calculated from fasting insulin and glucose measures using the HOMA-2 calculator.^49^

#### Continuous glucose monitoring

Glucose was also measured using continuous glucose monitoring [CGM]. CGMs (Medtronic, USA) recorded interstitial glucose levels through a probe inserted into subcutaneous abdominal fat, and glucose data was recorded in 5-min intervals and calibrated against 3–4 finger-prick blood glucose samples taken across the day. For the CGM data, the Mean Amplitude of Glycemic Excursions [MAGE]^50^ and the Continuous Overall Net Glycemic Action [CONGA]^51^ were calculated using the EasyGV V9.0.R2 macro in Microsoft Excel.^52^ The MAGE is defined as the average amplitude of glucose fluctuations with a magnitude of greater than 1 SD (42). The CONGA is defined as the glucose variability within short defined segments of a continuous time-series, e.g., 1 h.^51^

#### Gastric emptying

Gastric emptying was assessed by the ^13^C octanoic acid breath test.^37^^,^^38^^,^^39^ Participants were provided with a standardised test day breakfast enriched with 100mg of ^13^C octanoic acid stable isotope tracer, mixed into eggs as octanoic acid is readily solubilised in egg yolk.^35^^,^^36^ The ratio of ^13^C–^12^C in the breath was measured immediately before breakfast [-5mins] and at 15, 30, 45, 60, 75, 90, 120, 150, 180, 240, and 300 min after breakfast, and expressed as a percentage of dose per hour. Values for the cumulative percentage of ^13^CO_2_ recovered were fitted by the following model:

$$y=m{\left(1-e{-}^{kt}\right)}^{ß}$$

Where *y* is the cumulative percentage dose of ^13^CO_2_ recovered at time-point *t* (mins), and *m, k,* and *ß* are the estimated parameters. From this model, the gastric emptying half-time [*T-½*] and solid lag phase [*T-lag*] were calculated by non-linear regression.

#### Subjective measures

Participants also completed subjective measures of sleepiness, alertness, hunger, appetite, and fullness, every hour from waking for 16 h each test day [Figure 1B]. Participants completed the Karolinska Sleepiness Scale [KSS], which has been validated against electroencephalographic [EEG] activity measures.^53^ Participants also completed a subjective alertness scale, which has previously been shown to correlate with internal biological [i.e., circadian] time and with the duration of prior wakefulness.^54^ For subjective hunger, appetite, and satiety, participants completed a subjective visual analogue scale [VAS], which have been shown to have good reproducibility and validity for use in controlled feeding studies.^55^^,^^56^

### Quantification and statistical analysis

#### Power calculation and statistical analysis

Sample size estimation was based on previously published data by Morris et al.,^22^ which investigated the effects of 12 h circadian misalignment on early (∼2 h) thermic effect of feeding [TEF] and postprandial energy expenditure in a controlled laboratory intervention. Based on an expected difference of >1SD between TEF aligned to circadian vs. behavioral patterns, and for an alpha level of 5% and 90% power, a sample size of 13 was estimated. In anticipation of a 15% non-completion rate, the study aimed to run 15 participants through the experimental protocol.

Data were analyzed in IBM Statistical Package for the Social Sciences (IBM SPSS Statistics for Windows, Version 27.0. Armonk, NY, USA: IBM Corp) using a linear mixed model [LMM] with an compound symmetry covariance structure, with participants entered as random effects to account for within-person correlations on repeated measures over study days. Use of LMM prevented listwise deletion due to missing data. Where study day was the only dependent variable, this was entered as fixed effects [without interaction term] into the model, with participants entered as random effects. For outcome measures which had both study day and meal as dependent variables, these were entered as fixed effects with an interaction term, and participants as random effects. The accepted significance for all post-hoc comparisons following a statistically significant main effect were adjusted for multiple comparisons using the Bonferroni correction. Data from the KSS, alertness scale, and VAS, were analyzed using LMM in SAS (Version 9.4, NC, USA: SAS Institute Inc.). Data were assessed for normality by visual inspection of Q-Q normality plots and histograms of the residuals in SPSS, and calculation of z-scores for skewness and kurtosis (within ±1.96) where necessary. Data not following a normal distribution were log-transformed. The assumptions of linearity and homoscedasticity were checked against residuals plots of the residual and predicted values from the model. GraphPad Prism v9.1 (GraphPad Software 2021, La Jolla California, USA) was used for graphing of outcome measures.
