# Supplementary material for: Short-term changes in human metabolism following a 5-h delay of the light-dark and behavioral cycle
Source: iScience. 2024 Oct 11;27(11):111161. doi: 10.1016/j.isci.2024.111161 (PMC11607541; doi:10.1016/j.isci.2024.111161)
Supplement: Document S1. Figures S1–S3 and Tables S1 and S2 [file mmc1.pdf]

iScience, Volume *27*

Supplemental information

Short-term changes in human metabolism following a 5-h delay of the light-dark and behavioral cycle

Alan Flanagan, Leonie C. Ruddick-Collins, Barbara Fielding, Benita Middleton, Johanna von Gerichten, Michael Short, Victoria Revell, Jeewaka Mendis, Claus-Dieter Mayer, Peter J. Morgan, Alexandra M. Johnstone, and Jonathan D. Johnston

Figure S1: Sleep Parameters Estimated from Actigraphy

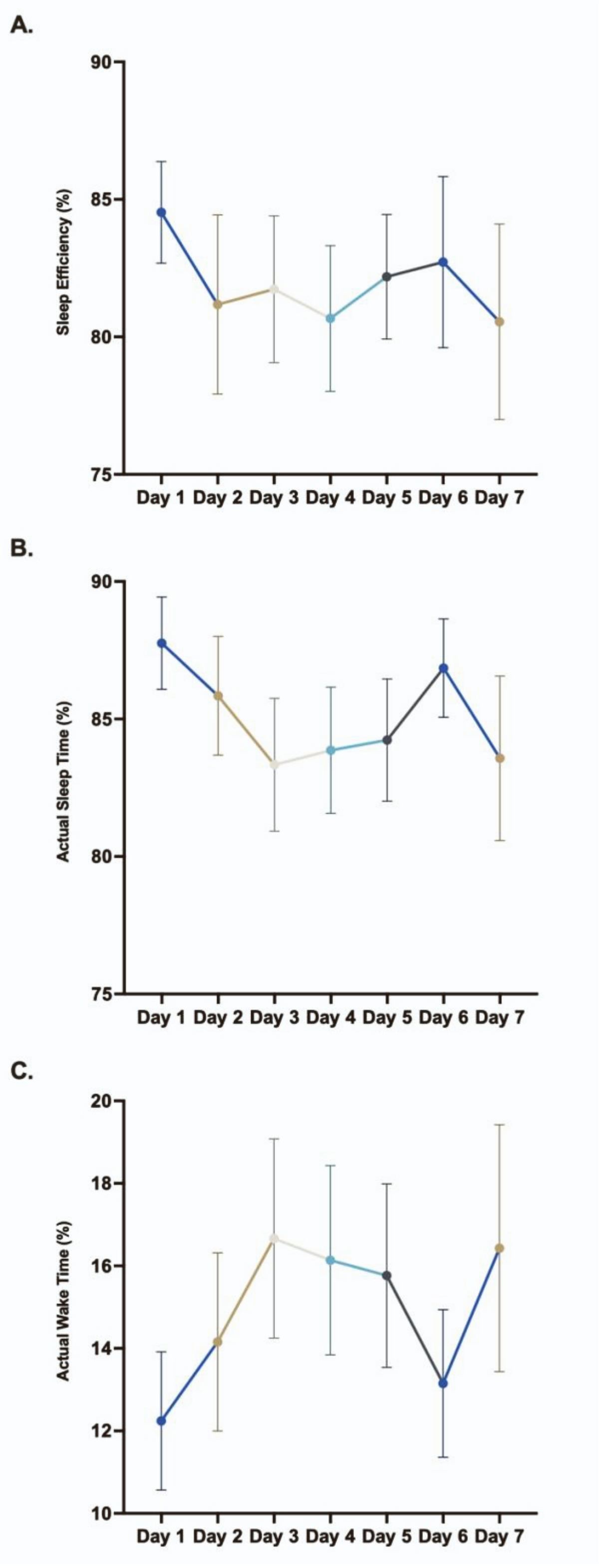

Figure S1. All data are presented as bars with mean and standard error. **A.** Sleep efficiency [as a percentage of total sleep time]; **B.** Actual sleep time [as a percentage of total sleep time]; **C.** Actual wake time [as a percentage of total sleep time]. Measured by actigraphy during the 8 h laboratory sleep opportunity before and after the phase shift. No significant differences in any of the sleep parameters measured [1-Way Repeated Measures ANOVA].

Figure S2: Postprandial Measures by Main Effect of Meal

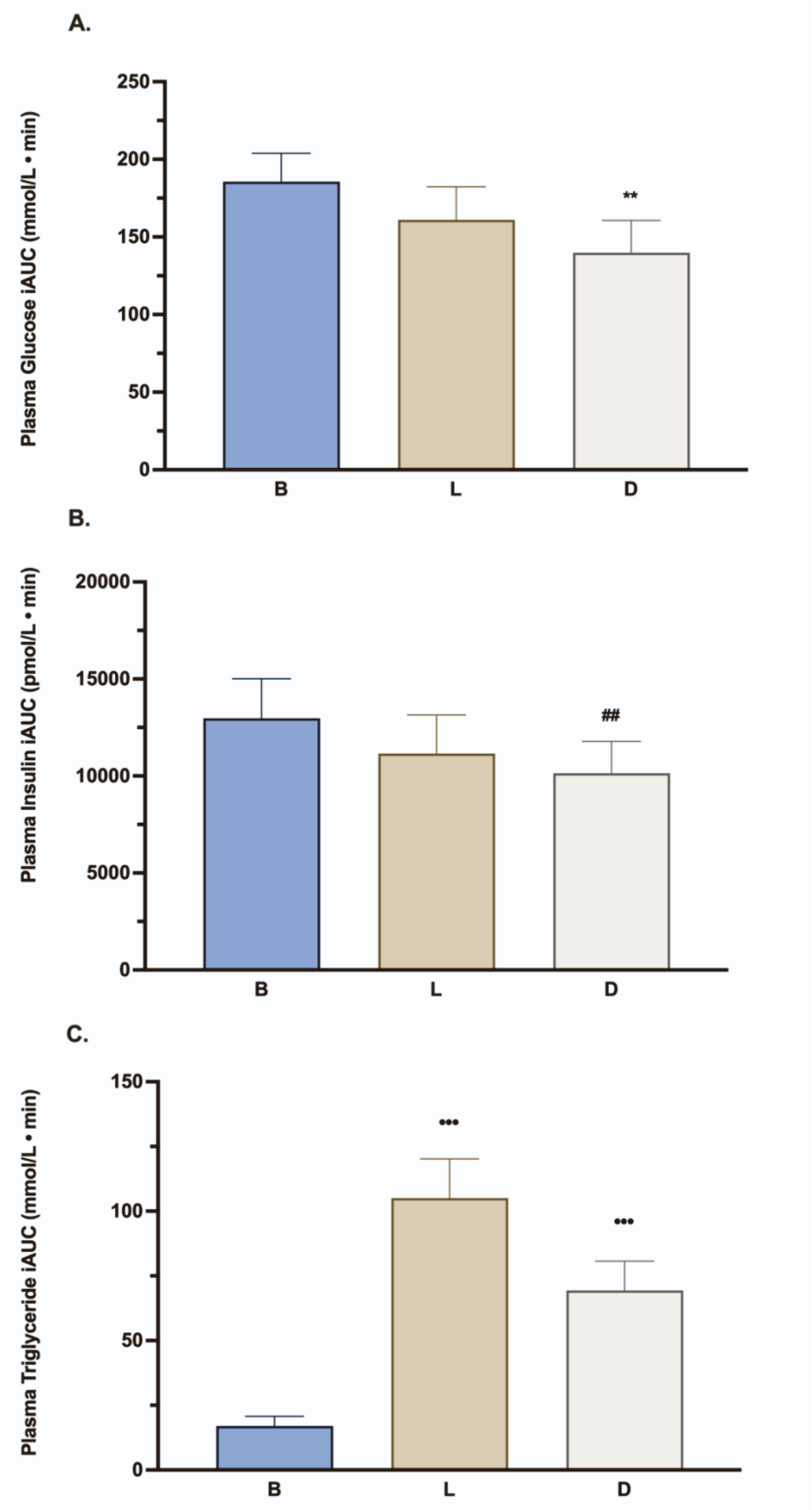

Figure S2. All data are presented as bars with mean and standard error. **A.** Postprandial plasma glucose incremental area under the curve [iAUC; mmol/L • min] for the main effect of meal. ** $p < 0.01$ for the difference in main effect of meal comparing dinner to breakfast. **B.** Postprandial plasma insulin iAUC [pmol/L • min] for the main effect of meal. ## $p < 0.01$ for the difference in main effect of meal comparing dinner to breakfast. **C.** Postprandial plasma triglycerides [TG] iAUC [mmol/L • min] for the main effect of meal; ••• $p < 0.001$ for the difference in plasma TG iAUC comparing lunch to breakfast, and comparing dinner to breakfast, respectively.

Figure S3: Subjective Hunger/Appetite Measures

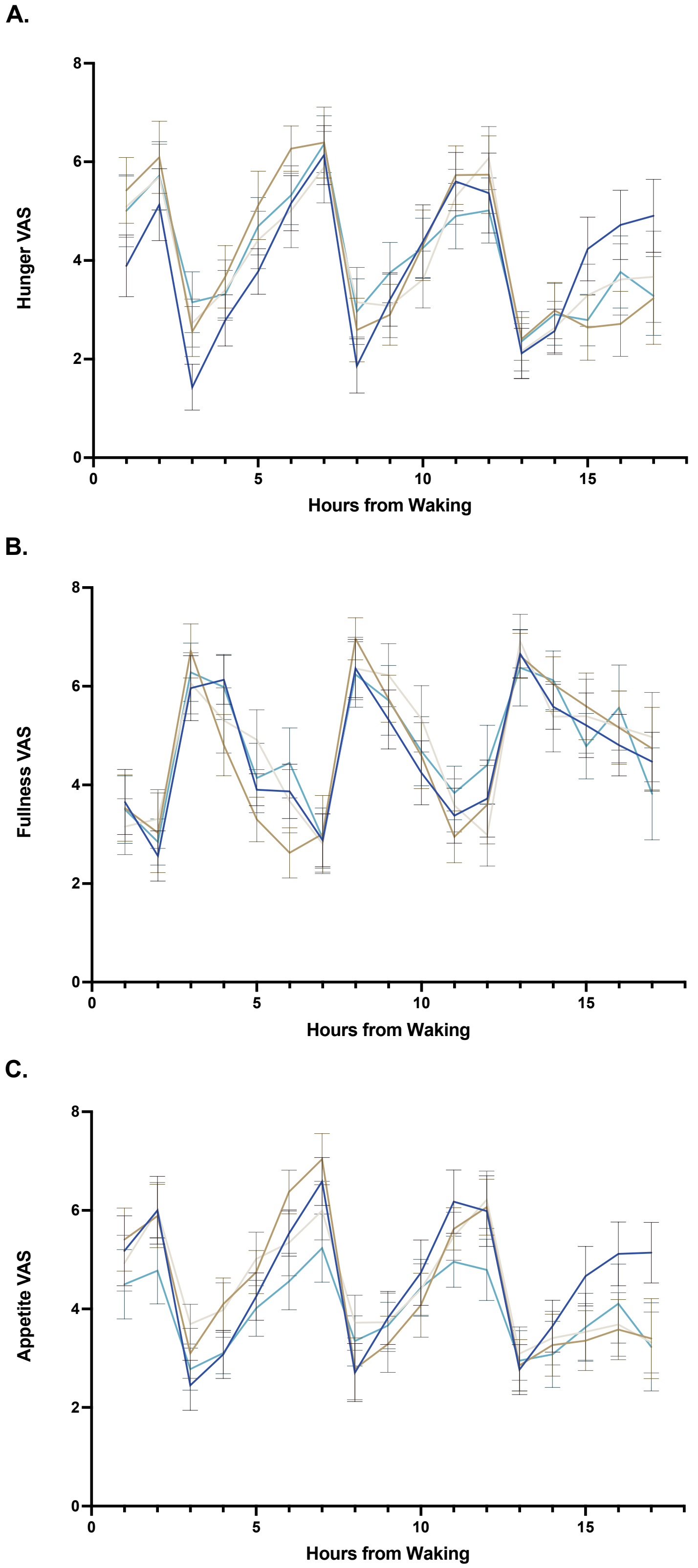

Figure S3. All data are presented as mean and standard error. Visual analogue scale measures of **A.** Appetite; **b.** Hunger, and; **C.** Fullness, between test days [Black = Day 2; Red = Day 3; Green = Day 5; Purple = Day 7].

Table S1: Dietary Energy, Macronutrient Content, and Food Weight

	Weight Food (g)	Energy (kJ)	Energy (kcal)	Fat (g)	Protein (g)	Carb (g)	%Fat	%Protein	%Carb
**Day 1**	2134.8 ± 114.6	9269.9 ± 387.3	2217.7 ± 92.7	83.8 ± 3.7	112.4 ± 4.6	255.9 ± 10.8	33.4 ± 0.2	20.6 ± 0.1	44.2 ± 0.1
**Day 2 (TEST DAY)**	2616 ± 162.2	12417 ± 558	2970.6 ± 133.5	112.2 ± 5.3	146.3 ± 6.5	348.3 ± 15.6	33.4 ± 0.2	20 ± 0	44.9 ± 0.1
**Day 3 (TEST DAY)**	2125.5 ± 125.9	9187.5 ± 432.9	2198 ± 103.6	83.3 ± 4.1	108.7 ± 5.1	257.4 ± 12.2	33.5 ± 0.2	20.1 ± 0	44.8 ± 0.1
**Day 4**	2256.5 ± 114.7	9300.3 ± 393.8	2225 ± 94.2	84.4 ± 3.9	109.9 ± 4.6	256.7 ± 10.9	33.5 ± 0.2	20.1 ± 0.1	44.2 ± 0.1
**Day 5 (TEST DAY)**	2147.9 ± 124.4	9210.9 ± 425.1	2203.6 ± 101.7	83.9 ± 4	108.6 ± 4.9	257.5 ± 12	33.7 ± 0.1	20.1 ± 0	44.7 ± 0.1
**Day 6**	2124.2 ± 123.2	9227.8 ± 420.7	2207.6 ± 100.6	83 ± 4.1	112 ± 4.9	255.3 ± 11.7	33.2 ± 0.2	20.7 ± 0.1	44.3 ± 0.1
**Day 7 (TEST DAY)**	2124 ± 131.4	9190.4 ± 454.5	2198.7 ± 108.7	83.3 ± 4.4	108.6 ± 5.2	257.6 ± 12.6	33.5 ± 0.1	20.1 ± 0	44.9 ± 0.1
**Test day 2 (3 meals only)**	2162.3 ± 125.5	9293.9 ± 431	2223.4 ± 103.1	83.8 ± 4.1	109.7 ± 5	261.6 ± 12.1	33.3 ± 0.2	20.1 ± 0	45 ± 0.2

Data presented as mean ± SEM. Data for Test Day 2 is presented as 3 meals only [bottom row] and including the additional extra evening snack to maintain a standardised 16 h fast overnight to breakfast [second row from top]. Food weight is in grams per day. Macronutrients are presented both as gram amounts and as a percentage of total daily energy intake.

Table S2: Energy Expenditure and Post-Prandial Data Between Study Days

	Day 2	Day 3	Day 5	Day 7
DLMO [24 h]	22:36 (± 00:31)	00:01 (± 00:34)	01:41 (± 00:34)	02:18 (± 00:35)
Wake Time to DLMO [h:min]	15 h 18min (± 30)	11 h 44min (± 30)	13 h 25min (± 30)	14 h 6min (± 32)
Whole Day EE [kJ]	5227.9 (± 200.3)	5179 (± 207.8)	5263.6 (± 211.9)	5289.0 (± 235.9)
Fasting RMR [kJ]	6636.4 (± 280.1)	6972.4 (± 314.7)	6923 (± 329.7)	6731.9 (± 278.4)
TEF [kJ]				
Breakfast	252.6 (± 23.9)	177.0 (± 23.9)	198.0 (± 27.8)	245.3 (± 35.3)
Lunch	278.7 (± 33.2)	213.7 (± 27.5)	234.0 (± 35.8)	276.1 (± 33.2)
Dinner	266.3 (± 28.7)	156.9 (± 28.6)	214.1 (± 41.0)	272.8 (± 35.9)
Gastric Emptying				
T-½ (mins)	318.1 (± 36.9)	408.5 (± 51.9)	336.7 (± 48.2)	293.3 (± 43.0)
T-Lag (mins)	223.6 (± 27.5)	269 (± 36.3)	237.1 (± 33.9)	211.2 (± 31.6)
Fasting Plasma Glucose (mmol/L)	5.6 (± 0.13)	5.3 (± 0.13)	5.3 (± 0.13)	5.4 (± 0.13)
Plasma Glucose iAUC (mmol/L • min)				
Breakfast	149.1 (± 25.8)	232.9 (± 25.8)	188.2 (± 25.8)	172.3 (± 25.8)
Lunch	139.0 (± 25.8)	174.5 (± 25.8)	169.2 (± 25.8)	161.1 (± 25.8)
Dinner	117.0 (± 25.8)	170.1 (± 25.8)	144.7 (± 25.8)	127.4 (± 25.8)
Continuous Glucose Monitor (mmol/L)				
MAGE	1.72 (± 0.56)	1.92 (± 0.60)	1.87 (± 0.48)	N/A
CONGA	5.32 (± 0.32)	5.18 (± 0.31)	5.30 (± 0.31)	N/A
Fasting Plasma Insulin (pmol/L)	26.0 (± 3.55)	25.2 (± 3.55)	27.1 (± 3.55)	24.5 (± 3.55)
Plasma Insulin iAUC (pmol/ • min)				
Breakfast	11874.8 (± 1957.4)	10847.5 (± 1957.4)	7851.9 (± 1957.4)	9244.5 (± 1957.4)
Lunch	8766.7 (± 1957.4)	10004.2 (± 1957.4)	7569.3 (± 1957.4)	6961.5 (± 1957.4)
Dinner	7488.5 (± 1957.4)	8267.3 (± 1957.4)	6326.5 (± 1957.4)	7370.6 (± 1957.4)
HOMA-IR	.43 (± .03)	.41 (± .02)	.44 (± .03)	.38 (± .02)
Fasting Plasma TG (mmol/L)	1.4 (± 1.18)	1.4 (± 1.18)	1.4 (± 1.18)	1.4 (± 1.18)
Plasma TG iAUC (mmol/L • min)				
Breakfast	12.7 (± 12.7)	14.1 (± 12.5)	14.8 (± 12.5)	20.7 (± 12.5)
Lunch	77.0 (± 12.5)	123.1 (± 12.5)	109.9 (± 12.5)	110.1 (± 12.5)
Dinner	54.0 (± 12.5)	81.4 (± 12.5)	80.3 (± 12.5)	68.8 (± 12.7)

Note: Data are presented as mean and standard error [SEM]. kJ = kilojoules; DLMO = Dim Light Melatonin Onset; EE = energy expenditure; RMR = resting metabolic rate; TEF = thermic effect of feeding; TG = triglycerides; MAGE = Mean Amplitude of Glycaemic Excursions; CONGA = Continuous Overall Net Glycaemic Action.
